# Meissner effect measurement of single indium particle using a customized on-chip nano-scale superconducting quantum interference device system

**DOI:** 10.1038/srep45945

**Published:** 2017-04-04

**Authors:** Long Wu, Lei Chen, Hao Wang, Xiaoyu Liu, Zhen Wang

**Affiliations:** 1 CAS Center for Excellence in Superconducting Electronics(CENSE), State Key Laboratory of Functional Materials for Informatics, Shanghai Institute of Microsystem and Information Technology (SIMIT), Chinese Academy of Sciences (CAS), Shanghai 200050, China; 2 University of Chinese Academy of Sciences, Beijing 100049, China; 3 School of Physical Science and Technology, Shanghai Tech University, Shanghai 200031, China

## Abstract

As many emergent phenomena of superconductivity appear on a smaller scale and at lower dimension, commercial magnetic property measurement systems (MPMSs) no longer provide the sensitivity necessary to study the Meissner effect of small superconductors. The nano-scale superconducting quantum interference device (nano-SQUID) is considered one of the most sensitive magnetic sensors for the magnetic characterization of mesoscopic or microscopic samples. Here, we develop a customized on-chip nano-SQUID measurement system based on a pulsed current biasing method. The noise performance of our system is approximately 4.6 × 10^−17^ emu/Hz^1/2^, representing an improvement of 9 orders of magnitude compared with that of a commercial MPMS (~10^−8^ emu/Hz^1/2^). Furthermore, we demonstrate the measurement of the Meissner effect of a single indium (In) particle (of 47 μm in diameter) using our on-chip nano-SQUID system. The system enables the observation of the prompt superconducting transition of the Meissner effect of a single In particle, thereby providing more accurate characterization of the critical field *H*_*c*_ and temperature *T*_*c*_. In addition, the retrapping field *H*_*re*_ as a function of temperature *T* of single In particle shows disparate behavior from that of a large ensemble.

The zero resistivity and Meissner effect are two of the most fundamental properties of superconductivity^1^, and both are crucial for identifying and studying superconducting materials. The zero resistivity can be simply characterized using an electrical transport measurement. The measurement of Meissner effect, which is sometime considered to be a more essential characterization and implies the zero resistivity, is indirect and therefore more complex. A commercial magnetic property measurement system (MPMS) based on a superconducting quantum interference device (SQUID) has been developed and widely used by many researchers^2^. Since such a system couples the sample to a distant SQUID through a pickup coil, a large sample size is required for appropriate characterization of the magnetic properties. However, as many emergent superconductivity phenomena appears on a smaller scale or at lower dimension^3^,^4^,^5^,^6^,^7^, such as the paramagnetic Meissner effect in small superconductors^3^ and the Little–Parks–de Gennes effect in ultra-small Al loops^6^. Therefore, a more sensitive method to measure the Meissner effect is highly desirable.

The sensitivity to magnetic moments of a SQUID can be significantly increased by shrinking down its washer size into nano-scale^8^,^9^,^10^,^11^. Because the nano-SQUID can be directly coupled to a small sample in the magnetic field^12^,^13^,^14^,^15^, it has made a great contribution to the field of nano-magnetism^16^,^17^,^18^,^19^. Recently, it was demonstrated that a nano-SQUID can be integrated on the tip of a scanning SQUID microscope to perform high-resolution imaging over a sample surface^20^,^21^,^22^,^23^,^24^. At the same time, a set of three-axis nano-SQUIDs was integrated on the same chip for directly performing the on-chip measurement of the vector magnetic moment^25^. In this paper, we demonstrate a simple method to measure the Meissner effect of a single indium (In) particle using a customized on-chip nano-SQUID system. The sensitivity in magnetic moments of our system reaches approximately 4.6 × 10^−17^ emu/Hz^1/2^, compared with that of a commercial MPMS (10^−8^ emu/Hz^1/2^)^2^, which represents an improvement of 9 orders of magnitude. Furthermore, our system observed a prompt superconducting transition of the Meissner effect, and thereby delivers a more accurate critical field *H*_*c*_ and critical temperature *T*_*c*_ compared with the measurement of a large ensemble by the MPMS. In addition, the behavior of the re-trapping field *H*_*re*_ as a function of temperature *T* for single In particle differs from that of a large ensemble. The disparate behavior is probably attributed to the large surface-to-volume ratio of the single particle leading to enhanced behavior of the surface states. We believe that our system will potentially lead to many promising discoveries that occur on a smaller scale or at a lower dimension in the emergent superconductivity field.

## Methods

### Nano-SQUID fabrication

**Figure 1:**
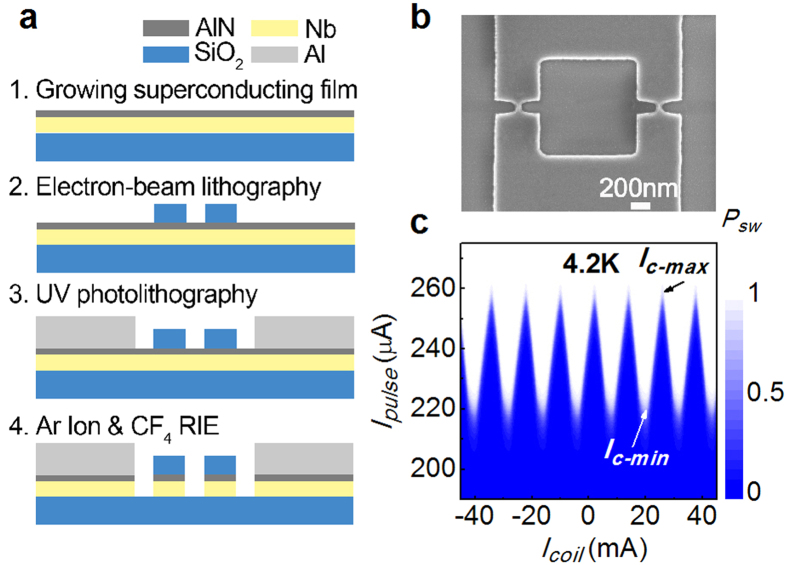
(**a**) Main steps of the nano-SQUID fabrication process. (**b**) SEM image of a typical SQUID; the loop area is 1 μm^2^, and the size of the junctions is 60 nm × 75 nm. (**c**) Flux modulation curve of the nano-SQUID.

The main fabrication process for our nano-SQUIDs is schematically illustrated in Fig. 1(a). The process starts with the growth of a 15 nm-thick niobium (Nb) film using direct-current magnetron sputtering on a Si substrate coated with a 400 nm-thick layer of silicon dioxide (SiO_2_). Then, a 2 nm-thick aluminum nitride (AlN) layer is grown *in-situ* on top of the Nb film to protect it from oxidation. Next, the nano-SQUID is patterned using a negative resist (XR1541-002) for electron beam lithography (EBL). After that, the nano-SQUID is electrically led out to contact pads using an ultraviolet(UV) photolithography step. Finally, a reactive ion etching (RIE) process is used to remove the unwanted part of the Nb/AlN film to form the nano-SQUID, as it is shown in Fig. 1(b). Compared to our previous process^26^, the using of the negative EBL resist avoids a troublesome lift-off step and increases the yields of SQUIDs. In Fig. 1(c), the critical current (*I*_*c*_) of a nano-SQUID is plotted as a function of the applied magnetic flux. The flux modulation depth (*FMD*) of the nano-SQUID is 14% at 4.2 K. Here, the *FMD* is defined as *FMD* = (*I*_*c-max*_ − *I*_*c-min*_)/*I*_*c-max*_, where the *I*_*c-max*_ and *I*_*c-min*_ represent *I*_*c*_ at the constructive and destructive quantum interference, respectively.

### On-chip SQUID measurement system

**Figure 2:**
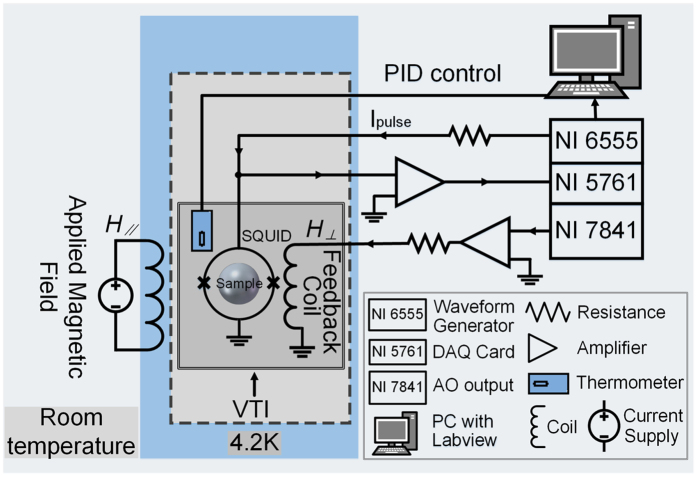
The schematic diagram of the on-chip nano-SQUID measurement system.

Our on-chip nano-SQUID measurement system is schematically illustrated in Fig. 2. The nano-SQUID chip was mounted on a chip carrier fixed onto the cold finger of a variable temperature insert (VTI). Above the chip carrier, a small hand-wound superconducting feedback coil was placed to flux bias the SQUID. The entire VTI was inserted into the middle of a superconducting magnet, which can provide a large parallel magnetic field *H*_*//*_. The single particle was placed on the chip near the SQUID loop to provide optimal coupling.

Because a the planar nano-SQUID usually shows a hysteretic current–voltage (*I*-*V)* curve, we used a pulsed current bias method to obtain the readout such that the SQUID was reset to zero bias at the end of each current pulse. We also assembled a field-programmable-gate-array (FPGA) based SQUID readout system to perform a fast measurement as illustrated in Fig. 2. The pulsed current bias method works as follows. A constant number *N*_*pulse*_ of current pulses of amplitude *I*_*pulse*_ was sent to a nano-SQUID using a waveform generator (NI PXIe-6555) in series with a large resistance *R* = 4 kΩ. The width of generated pulse is 1.6 μs with a repetition frequency of 250 kHz. Then, the corresponding voltage pulses *V*_*sw_i*_ across the SQUID was collected using a high speed analog data acquisition card (DAQ NI 5761) in connection with a room-temperature preamplifier. The voltage pulse number *N*_*sw*_ was counted by the *V*_*sw_*i_ greater than a threshold voltage *V*_*th*_. The on-board FPGA circuit was programmed to detect the corresponding voltage pulse and calculate the switching probability *P*_*sw*_ = *N*_*sw*_/*N*_*pulse*_ and average pulse height *V*_*sw*_ = Σ*V*_*sw_*i_/*N*_*pulse*_. Then, based on the *P*_*sw*_ or *V*_*sw*_, the FPGA locked the SQUID by tuning the analog output current (NI PXI-7841R) to the feedback coil to compensate for the flux variation. In this manner, the entire SQUID feedback process was performed by the FPGA without talking to the computer. The current pulse could be as short as 500 ns and was limited by the bandwidth of the wires connected to the SQUID.

## Results and Discussion

### Characterization of system noise

**Figure 3:**
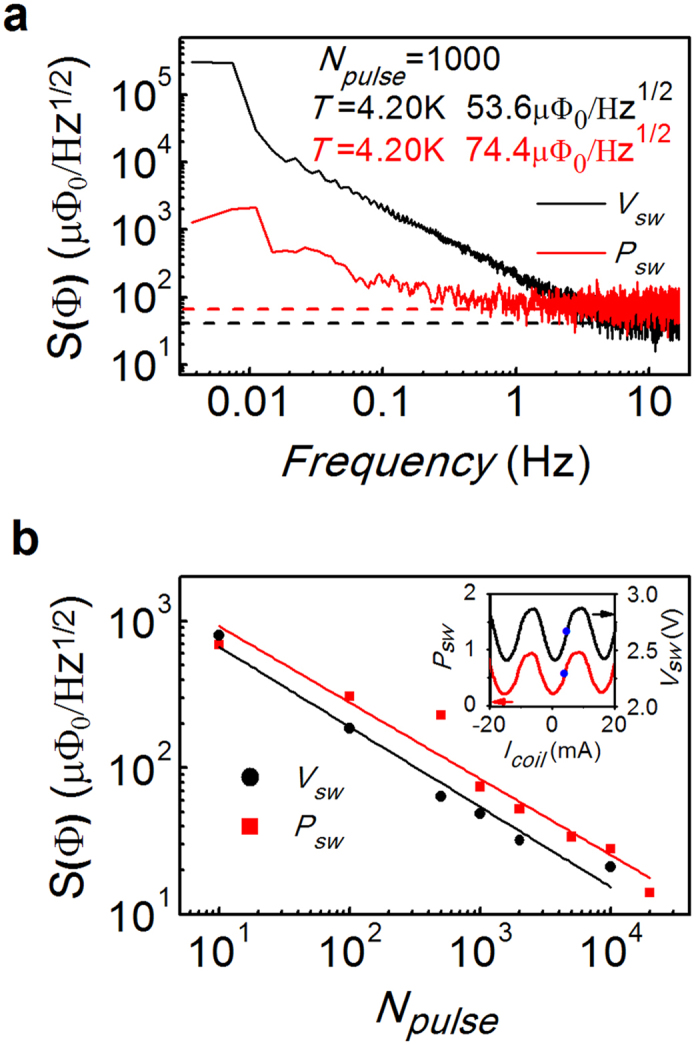
(**a**) Flux noise density spectra obtained by measuring *P*_*sw*_ (red curve) and *V*_*sw*_ (black curve) respectively for *N*_*pulse*_ = 1000. (**b**) White flux noise density for various number of pulses *N*_*pulse*_ by measuring *P*_*sw*_ (red squres) and *V*_*sw*_ (black dots). The solid lines are linear fits to a function of *N*_*pulse*_^1/2^. The inset shows both *P*_*sw*_ (red) and *V*_*sw*_ (black) of the SQUID as a function of the feedback coil current and the working points (blue points) where the flux noise density spectra were measured.

Because a low-noise measurement system is essential for characterizing the magnetic properties of small samples, we evaluated the noise performance of our on-chip nano-SQUID measurement system in terms of both *P*_*sw*_ and *V*_*sw*_, as shown in Fig. 3. The inset of Fig. 3(b) demonstrates that both *P*_*sw*_ and *V*_*sw*_ were periodically modulated by *I*_*coil*_ at a bias of *I*_*pulse*_ = 239 μA. In order to characterize the flux noise, the flux bias of the SQUID was fixed at *I*_*coil*_ = 3.75 mA, as indicated by the the blue point in the inset of Fig. 3(b). By measuring *P*_*sw*_ and *V*_*sw*_ as a function of time *t* and converting the data into the frequency domain by using of a fast Fourier transform, the noise density spectra *S*_*Psw*_ and *S*_*Vsw*_ were obtained. As observed in the Fig. 3(a), the flux noise density spectrum *S(Ф*)_*Psw(Vsw*)_ = *Ф*_*0*_/*I*_*coil*_^*period*^ × δ*Ф*/δ*P*_*sw*_(δ*V*_*sw*_) × *S*_*Psw*_(*S*_*Vsw*_), where *I*_*coil*_^*period*^ = 13.9 mA corresponds to the current in the feedback coil generating a single flux quanta *Ф*_0_ to the SQUID, and δ*Ф/*δ*P*_*sw*_(δ*V*_*sw*_) is the inverse of the derivative at the blue point of the *P*_*sw*_ (*V*_*sw*_) modulation curve in the inset of Fig. 3(b). For *N*_*pulse*_ = 1000, the white flux noise was determined to be 74.4 and 53.6 μΦ_0_/Hz^1/2^ at 4.2 K by measuring *P*_*sw*_ and *V*_*sw*_ respectively. Although the white flux noise determined by measuring *P*_*sw*_ was slightly greater than that determined by measuring *V*_*sw*_, the low-frequency part obtained by measuring *P*_*sw*_ was much better. Therefore, a lower noise floor was achieved by measuring *P*_*sw*_ for the slow measurement such as the magnetization curve of sweeping the magnetic field. The flux noise density on the log scale also improved linearly upon increasing *N*_*pulse*_^*1/2*^ as observed in Fig. 3(b). For N = 10^4^, the white flux noise determined by measuring *P*_*sw*_ reached 28 μФ_0_/Hz^1/2^, with a corresponding sensitivity in magnetic moments of approximately 4.6 × 10^−17^ emu/Hz^1/2^ 8,27,28. Compared with the value for a commercial MPMS system (Quantum Design MPMS-3) of 1.0 × 10^−8^ emu/Hz^1/2^, our on-chip SQUID measurement system showed an improvement of 9 orders of magnitude.

In principle, the intrinsic flux noise of nano-SQUIDs may approach the quantum limit because of its ultra-low inductance^9^. Several state-of-the-art nano-SQUIDs have already achieved the intrinsic flux noise below 50 nФ_0_/Hz^1/2^, such as the Pb nano-SQUID-on-tip^29^, the YBCO nano-SQUID made by the focused ion beam^30^,^31^, the Al 3D nano-SQUID made by the shadow evaporation^32^, etc. Recently, we also made Nb 3D nano-SQUID with the intrinsic flux noise of 340 nФ_0_/Hz^1/213^. The planar Nb nano-SQUID in Fig. 1(b) do have a large intrinsic flux noise because of its large inductance, but can be operated in a high parallel magnetic field^12^. However, the system noise of 28 μФ_0_/Hz^1/2^ has been limited by the room-temperature amplifier and not reached its intrinsic noise level yet. Therefore, the improvement of another two orders of magnitude in the sensitivity of magnetic moments can be optimistically expected.

### On-chip Meissner effect measurement of a single In particle

**Figure 4:**
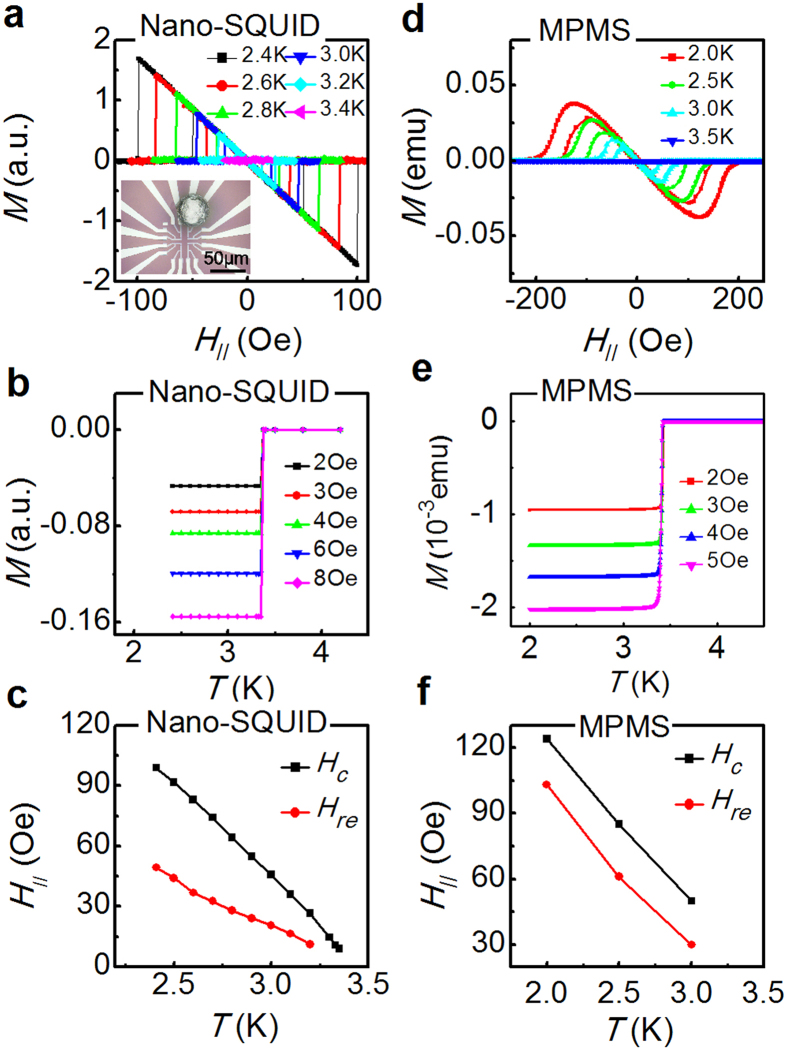
(**a**) *M* as a function of applied magnetic field *H*_*//*_ of a single In particle measured by the on-chip SQUID system at various temperatures. (**b**) *M* as a function of *T* of a single In particle measured by the on-chip SQUID system at various *H*_*//*_. (**c**) *H*_*c*_ (black squares) and *H*_*re*_ (red dots) as a function of the *T* from Figure 4(a). (**d**) The *M-H*_*//*_ curve of a large ensemble of In particles (approximately 61,000 particles) measured by the MPMS at various *T*. (**e**) *M-T* curve of a large ensemble of In particles measured by the MPMS system. (**f**) *H*_*c*_ (black squares) and *H*_*re*_ (red dots) as a function of *T* from Figure 4(d).

For demonstration, we measured the Meissner effect of a single indium (In) particle. The single In particle was placed on the SQUID chip, as shown in the inset of Fig. 4(a), and cooled to low temperature by the VTI. The SQUID was locked at its working point by the FPGA-controlled readout system as described in the section of Methods. Then, by slowly sweeping the magnetic field *H*_*//*_, the magnetic flux variation generated by the Meissner effect of the single In particle was directly picked up by the on-chip SQUID. As illustrated in Fig. 4(a), by ramping up the *H*_*//*_, the sample switched from a diamagnetic branch to zero magnetization at the field *H*_*c*_, which indicated that the In particle switched from the superconducting to normal state. As the field ramped down, the In particle was re-trapped back to the diamagnetic branch at a lower field *H*_*re*_. The magnetization *M* as a function of temperature *T*, at various *H*_*//*_, is also plotted in Fig. 4(b). M generated by the superconducting Meissner effect switches back to the normal state at *T* = *T*_*c*_. In Fig. 4(c), the *H*_*c*_ and *H*_*re*_ are plotted as a function of *T*.

For comparison, Fig. 4(d,e) present Meissner effect measurements of the In particles in a large ensemble (approximately 61,000 particles with diameters of 30–50 μm) by using of the commercial MPMS. In Fig. 4(d), the *M-H* measurement of the large ensemble clearly shows a smeared-out transition between the superconducting and normal state. The transition of *M* at *T*_*c*_ is also broadened. In contrast, the single In particle measured by the on-chip SQUID system showed a much prompt transition, which is expected for a Type I superconductor. The broadened or smeared–out transition observed in the particle ensemble might originate from the variation of the individual particles or a clustering effect that distorts the local magnetic field for each particle. Therefore, the on-chip SQUID measurement delivers a more accurate characterization at *H*_*c*_ and *T*_*c*_. Moreover, the *H*_*re*_ -*T* curve of a single In particle in Fig. 4(c) has a different slope than the *H*_*c*_ –*T* curve. For the large ensemble, the slope of the *H*_*re*_*-T* curve is approximately equal to that of the *H*_*c*_*-T* curve. *H*_*re*_ is believed to be related to a super-cooling process^33^,^34^, in which the nucleation center near the surface prevents the forming of superconducting phase below *H*_*c*_ by lowering the magnetic field. Furthermore, the super-cooling effect is supposed to decrease as *T* approaches *T*_*c*_ because the coherence length and the penetration depth diverges at *T*_*c*_. Therefore, the disparate *H*_*re*_*-T* behavior of a single In particle from the large ensemble reveals more information about the surface state of small superconductors. Therefore, we believe that our system is a powerful tool to study the emergent phenomena of superconductivity that occur on a smaller scale or at a lower dimension.

## Conclusion

In order to measure the Meissner effect of small superconductors, we developed a simple process for fabricating planar Nb nano-SQUIDs based on a negative EBL resist and constructed an on-chip nano-SQUID measurement system based on a current pulse biasing method and FPGA-controlled feedback. The noise performance of the system reached S(Ф)_*Psw*_ = 28 μФ_0_/Hz^1/2^ (approximately 4.6 × 10^-17^ emu/Hz^1/2^), which represents an improvement of 9 orders of magnitude compared with that of a commercial MPMS system (1.0 × 10^−8^ emu/Hz^1/2^). Furthermore, we demonstrated the measurement of the Meissner effect of a single In particle using our on-chip nano-SQUID measurement system. The system enabled the observation of the prompt Meissner effect transition of a single In particle and delivered a more accurate characterization at *H*_*c*_ and *T*_*c*_. In addition, the disparate behavior of retrapping field *H*_*re*_ as a function of temperature *T* of a single In particle compared with that of a large ensemble clearly indicate the presence of surface nucleation center only by the on-chip nano-SQUID measurement. Therefore, we believe that our system is a powerful tool to study the emergent phenomena of superconductivity that occur on a smaller scale or at a lower dimension.

## Additional Information

**How to cite this article**: Wu, L. *et al*. Meissner effect measurement of single indium particle using a customized on-chip nano-scale superconducting quantum interference device system. *Sci. Rep.* **7**, 45945; doi: 10.1038/srep45945 (2017).

**Publisher's note:** Springer Nature remains neutral with regard to jurisdictional claims in published maps and institutional affiliations.
